# Spontaneous pseudoaneurysm of a profunda femoris artery perforator in a pediatric patient

**DOI:** 10.1016/j.radcr.2026.04.091

**Published:** 2026-05-21

**Authors:** Brian L. Han, Osahon O. Obanor, Auh Whan Park, Elliot S. Rinzler, Alexandra Callan, Sheena Pimpalwar

**Affiliations:** a Section of Pediatric Radiology, Department of Radiology, University of Texas Southwestern Medical Center, Dallas, TX, USA; b University of Texas Southwestern Medical School, Dallas, TX, USA; c Section of Musculoskeletal Oncology, Department of Orthopaedic Surgery, University of Texas Southwestern Medical Center, Dallas, TX, USA; d Children’s Medical Center Dallas, Pediatric Interventional Radiology, Dallas, TX, USA

**Keywords:** Pseudoaneurysm, Profunda femoris artery, Pediatric vascular abnormality, CT angiography, Ultrasound, Magnetic resonance imaging, Endovascular embolization

## Abstract

Profunda femoris artery pseudoaneurysms are rare in pediatric patients, particularly in the absence of trauma or prior intervention. We report a spontaneous pseudoaneurysm arising from a perforating branch of the profunda femoris artery in a previously healthy 14-year-old boy who presented with acute thigh pain, swelling, and anemia. Initial ultrasound demonstrated a large intramuscular hematoma without internal Doppler flow. Subsequent contrast-enhanced CT angiography identified an actively enhancing pseudoaneurysm with active extravasation. MRI was performed to further characterize the lesion and exclude an underlying soft-tissue mass or vascular malformation. The patient was successfully treated with urgent endovascular coil embolization followed by surgical evacuation of the residual hematoma. This case highlights the value of multimodality imaging in the diagnosis and management of spontaneous pediatric vascular hemorrhage.

## Introduction

Pseudoaneurysms of the profunda femoris artery (PFA) are uncommon vascular lesions that most often occur after trauma, orthopedic injury, or iatrogenic arterial insult in adults [[1], [2], [3]]. Pediatric cases are rarely reported, particularly in the absence of a clear precipitating event [4]. Although connective tissue disorders and vasculitic processes may predispose younger patients to spontaneous pseudoaneurysm formation, many clinicians may not initially consider this diagnosis in a child presenting with acute thigh pain or swelling [[5], [6], [7]].

We report a spontaneous pseudoaneurysm arising from a perforating branch of the PFA in a previously healthy 14-year-old boy and highlight the diagnostic value of multimodality imaging and successful endovascular management.

## Case presentation

A previously healthy 14-year-old boy presented to an outside hospital with acute onset left thigh pain and progressive swelling while seated at home. In the emergency department, he was tachycardic, pale, diaphoretic, and experienced a syncopal episode. Laboratory evaluation demonstrated acute blood loss anemia (hemoglobin 7.1 g/dL, hematocrit 20.2%), leukocytosis, and markedly elevated creatine kinase, with normal coagulation studies and no significant inflammatory marker elevation.

Physical examination revealed a large tender mass involving the proximal medial thigh with associated edema. Distal perfusion was preserved, and orthopedic evaluation found no evidence of compartment syndrome at presentation.

Initial ultrasound and CT performed at the outside facility demonstrated a large intramuscular hematoma with concern for associated pseudoaneurysm. After transfusion of one unit of packed red blood cells, the patient was transferred to our tertiary pediatric center for higher-level care and urgent further management.

## Imaging findings

### Ultrasound

**Fig. 1:**
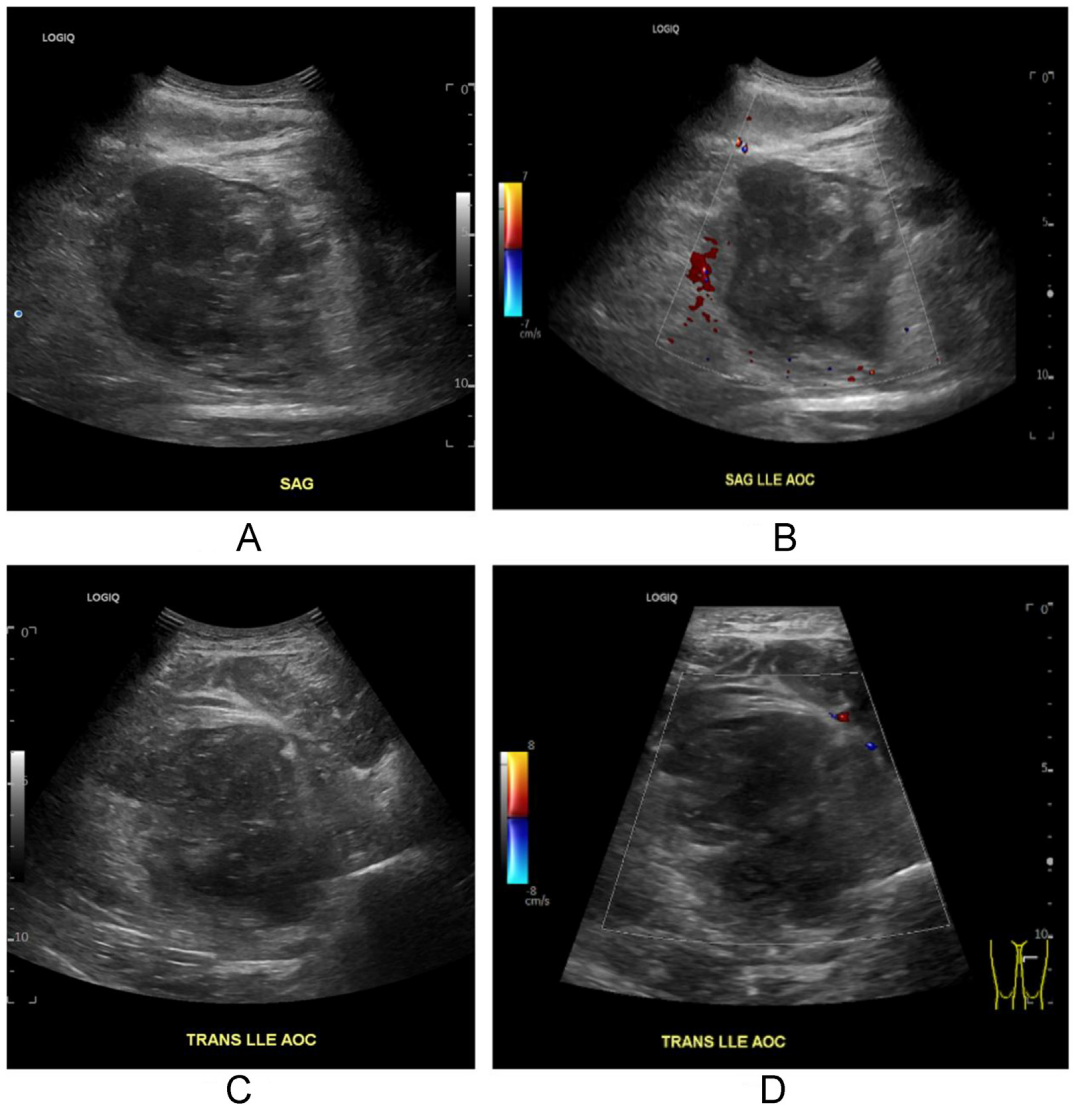
Sagittal grayscale (A) and color Doppler (B), and transverse grayscale (C) and color Doppler (D) ultrasound images of the left thigh demonstrate a heterogeneous intramuscular hematoma within the medial thigh musculature. No internal Doppler flow is identified within the hematoma.

Ultrasound of the left thigh demonstrated a heterogeneous intramuscular mass within the medial thigh musculature measuring approximately 6.3 × 6.5 × 7.0 cm, consistent with hematoma. No internal Doppler flow was identified. Visualized arterial and venous structures were patent without thrombosis (Fig. 1).

### CT angiography

**Fig. 2:**
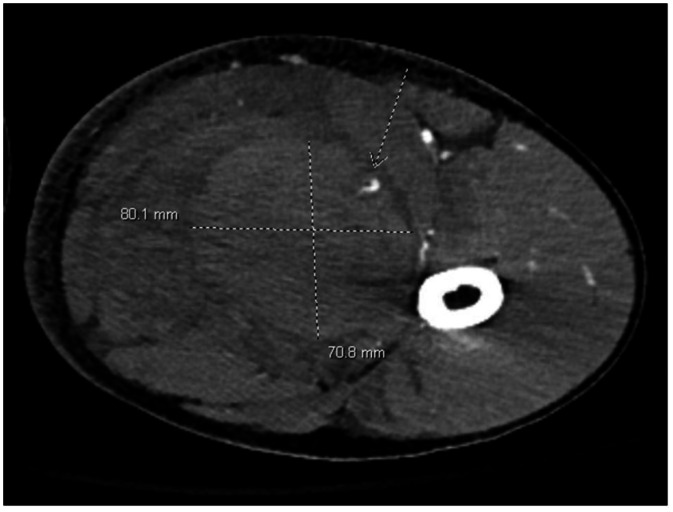
Axial contrast-enhanced CT image of the left thigh demonstrates a large intramuscular hematoma within the adductor compartment. A focal enhancing pseudoaneurysm is present centrally (dotted arrow), with associated compression of the adjacent femoral vein.

Contrast-enhanced CT of the left thigh demonstrated a large hyperdense intramuscular hematoma within the adductor compartment measuring approximately 9.1 × 7.2 × 10.0 cm with surrounding edema. Within the hematoma was a focal enhancing pseudoaneurysm arising from a perforating branch of the left profunda femoris artery, with active contrast extravasation. Associated mass effect compressed the adjacent femoral vein (Fig. 2).

### Magnetic resonance imaging

**Fig. 3:**
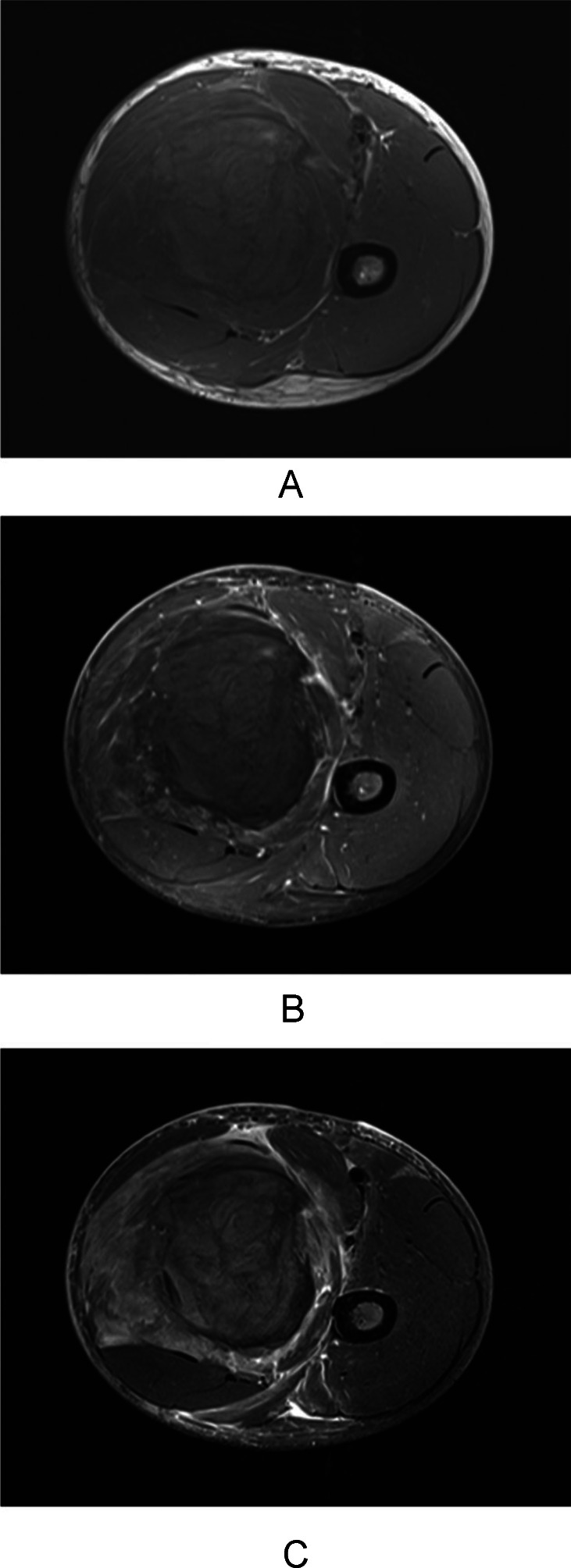
Axial T1-weighted (A), axial postcontrast fat-suppressed T1-weighted (B), and axial T2-weighted STIR (C) MRI images demonstrate a heterogeneous intramuscular hematoma within the left adductor compartment with internal blood products and extensive surrounding edema. No suspicious internal enhancement is identified.

MRI of the left thigh was obtained to exclude an underlying soft-tissue neoplasm or vascular malformation. Imaging demonstrated a heterogeneous hemorrhagic mass in the left adductor compartment measuring 7.5 × 8.8 × 12.6 cm with mixed T1 and T2 signal characteristics, scattered intrinsic T1 hyperintense foci consistent with blood products of varying age, minimal internal enhancement, and extensive surrounding edema. Findings were most consistent with a large organizing intramuscular hematoma (Fig. 3).

### MR angiography

**Fig. 4:**
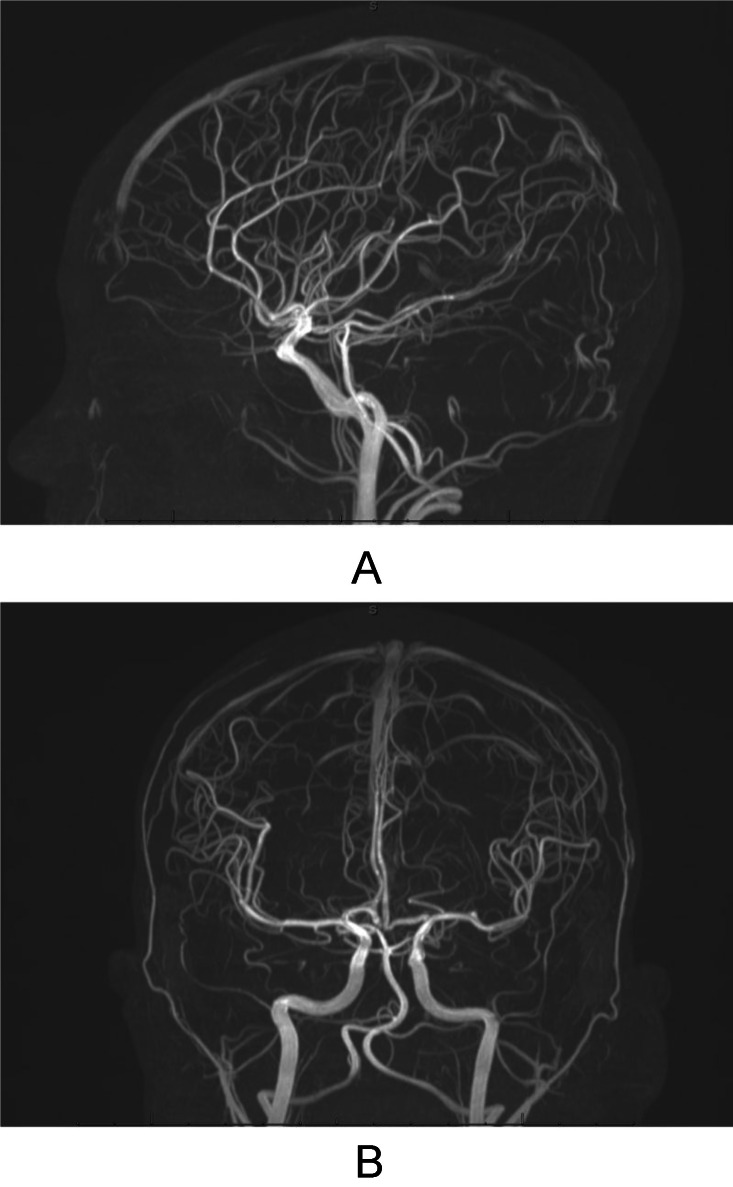
Sagittal (A) and coronal (B) time-of-flight MR angiography images of the intracranial circulation demonstrate no aneurysm, stenosis, or other vascular abnormality.

Intracranial time-of-flight MR angiography was subsequently performed to screen for associated vascular abnormalities in the setting of possible connective tissue disease. No aneurysm, stenosis, or other cerebrovascular abnormality was identified (Fig. 4).

## Treatment and outcome

### Endovascular embolization

Following transfer, the patient remained clinically concerning for ongoing hemorrhage despite fluid resuscitation and blood transfusion. Given persistent anemia, tachycardia, and imaging evidence of active bleeding with risk for evolving compartment syndrome, emergent angiography and endovascular intervention were performed.

**Fig. 5:**
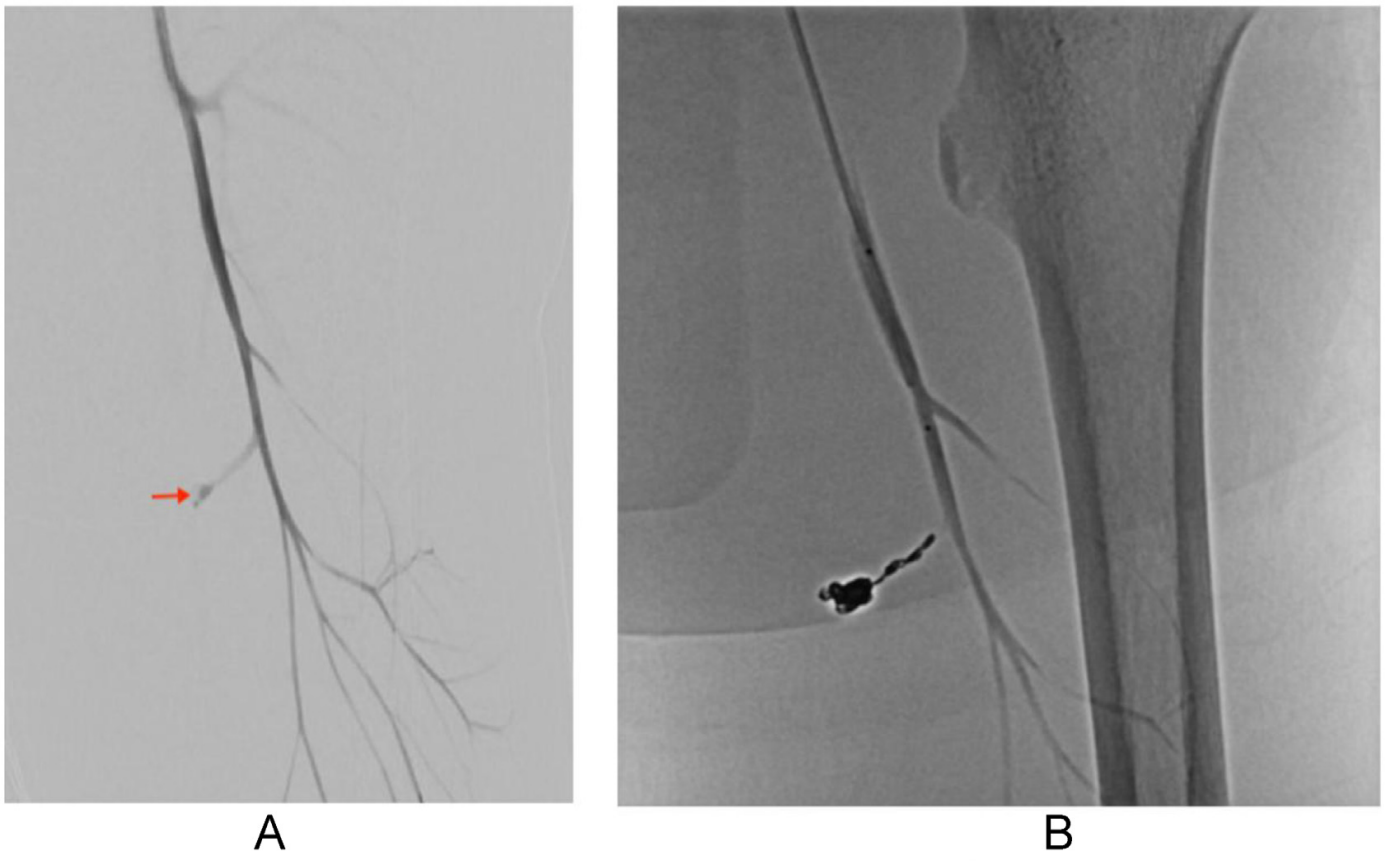
(A) Pre-embolization digital subtraction angiography of the left profunda femoris artery demonstrates an actively extravasating pseudoaneurysm arising from a perforating branch (arrow). (B) Post-embolization angiography demonstrates complete occlusion of the pseudoaneurysm and feeding vessel without residual extravasation.

Selective left lower extremity angiography confirmed an actively extravasating pseudoaneurysm arising from a perforating branch of the left profunda femoris artery (Fig. 5). Coil embolization of the pseudoaneurysm and feeding branch was successfully performed, with completion angiography demonstrating complete occlusion and no residual extravasation. The patient tolerated the procedure without immediate complication.

Although there were no clinical signs of compartment syndrome after embolization, the patient continued to experience substantial thigh pain, swelling, and difficulty ambulating. Surgical evacuation of the residual hematoma was therefore performed, yielding more than 1 liter of coagulated blood. No active bleeding, infection, or underlying mass was identified. Pathologic evaluation was consistent with organized hematoma.

Postoperatively, the patient had marked improvement in pain, swelling, and mobility. Hematologic evaluation for common bleeding disorders was unrevealing. Genetic testing for connective tissue disease was recommended but not completed. The patient was discharged on postoperative day 10 and remained asymptomatic at 3-month follow-up without imaging evidence of recurrence.

## Discussion

Pseudoaneurysms involving the profunda femoris artery are uncommon lesions that are most often associated with trauma, orthopedic injury, or iatrogenic vascular insult in adults [[1], [2], [3]]. Reports in pediatric patients are rare, particularly in the absence of a clear precipitating event [4].

To our knowledge, a spontaneous pseudoaneurysm arising from a perforating branch of the PFA in an otherwise healthy pediatric patient has only rarely been described in the available literature.

When a child or adolescent presents with acute thigh pain, swelling, anemia, or a rapidly enlarging intramuscular mass without obvious trauma, vascular injury should remain in the differential diagnosis. Additional considerations include soft-tissue neoplasm, vascular malformation, coagulopathy, infection, and compartment syndrome. Connective tissue disorders such as vascular Ehlers-Danlos syndrome and inflammatory vasculitides may also predispose to spontaneous arterial wall injury and pseudoaneurysm formation [[5], [6], [7]].

This case also highlights the limitations of ultrasound as an initial imaging modality. Although sonography identified a large hematoma, internal Doppler flow was not detected. False-negative Doppler evaluation may occur with partial thrombosis, slow or intermittent flow, deep lesion location, surrounding mass effect, or technical limitations. In this setting, contrast-enhanced CT angiography rapidly established the diagnosis by demonstrating the pseudoaneurysm, active extravasation, and local mass effect, thereby facilitating urgent intervention.

MRI provided complementary value by excluding an enhancing soft-tissue mass or high-flow vascular malformation and by further characterizing the hemorrhagic lesion. Subsequent intracranial time-of-flight MR angiography was performed as a screening examination for associated vascular abnormalities in the setting of possible underlying connective tissue disease.

Management options for peripheral pseudoaneurysms include ultrasound-guided thrombin injection, endovascular embolization, and open surgical repair [8,9]. Endovascular therapy is often favored when technically feasible because it offers rapid hemorrhage control with lower procedural morbidity [9]. In this case, coil embolization achieved definitive hemostasis, while subsequent hematoma evacuation provided symptomatic relief and improved mobility.

This report has limitations. No definitive underlying etiology was identified, and subtle unrecognized trauma or exertional microinjury cannot be completely excluded. In addition, recommended genetic evaluation for connective tissue disease was not completed. Despite these limitations, the case underscores the importance of maintaining suspicion for occult vascular injury in atraumatic pediatric soft-tissue hemorrhage and demonstrates the value of a multimodality imaging approach paired with timely minimally invasive treatment.

## Conclusion

Profunda femoris artery pseudoaneurysm is an uncommon cause of acute thigh swelling and anemia in pediatric patients, particularly without preceding trauma. This case demonstrates that occult vascular hemorrhage should be considered in atraumatic intramuscular masses. Ultrasound may be nondiagnostic, while CT angiography can rapidly establish the diagnosis and guide urgent treatment. Endovascular embolization offers an effective minimally invasive management option, with surgical evacuation reserved for persistent symptomatic hematoma or compartment-related concerns.

## Patient consent

Written informed consent was obtained from the patient’s legal guardian for publication of this case report and the accompanying images.
